# Can Neutrophil-to-Lymphocyte Ratio, Platelet Volume, and Platelet Distribution Width Be Used as Indicators of Delirium?

**DOI:** 10.3390/jcm13247532

**Published:** 2024-12-11

**Authors:** Bilge Tuncer, Fulya Çelik, Burak Çelik, Ezgi Erkılıç

**Affiliations:** 1 Department of Anesthesiology and Reanimation, Ankara Bilkent City Hospital, Ankara 06800, Turkey; eerkilic72@yahoo.com; 2 Department of Anesthesiology and Reanimation, Ankara Etlik City Hospital, Ankara 06010, Turkey; lfulyaeser@hotmail.com; 3 Department of Ear Nose Throat Surgery, Ankara Bilkent City Hospital, Ankara 06800, Turkey; burak-celik70@hotmail.com

**Keywords:** postoperative delirium, children, general anesthesia, neutrophil/lymphocyte ratio

## Abstract

**Background/Objectives:** Postoperative delirium is a frequent complication in children undergoing general anesthesia. It has been suggested that inflammation and oxidative stress contribute to the pathophysiology of delirium. The aim of our study was to investigate the relationship between inflammatory markers and delirium. **Methods:** A total of 221 children in the ASA 1–3 risk group who underwent adenoidectomy, tonsillectomy, and/or ventilation tube application were included in our single-center, prospective, and observational study approved by the Ethics Committee, numbered E1-23-3197. Patients whose parental consent was obtained were either premedicated with oral midazolam preoperatively or taken to surgery without premedication, depending on the anesthesiologist’s preference. After induction with sevoflurane, rocuronium and fentanyl were administered intravenously. Sevoflurane and infusion of remifentanil were administered for maintenance. At the end of the operation, patients were transferred to the recovery unit and followed up for 30 min. They were evaluated with the Face, Legs, Activity, Cry, and Consolability (FLACC) Scale and Pediatric Anesthesia Emergence Delirium (PAED) Scale. **Results:** Delirium was observed in 80 patients. There was no significant difference between patients with and without delirium in terms of neutrophil/lymphocyte ratio, mean platelet volume, or platelet distribution volume. Lymphocyte count, PAED score, and FLACC score were statistically higher in patients with delirium (W = 4407.5, *p*-value = 0.006997; W = 0, *p*-value < 2.2 × 10^−16^; W = 9489, *p*-value < 2.2 × 10^−16^, respectively). **Conclusions:** No statistically significant relationship was found between hematologic inflammatory markers and delirium. Controlled studies with larger sample sizes are needed to determine whether these markers have strong predictive value.

## 1. Introduction

Postoperative delirium is one of the most common postoperative complications, particularly among the elderly and pediatric populations. It generally presents as a complex of behavioral disturbances and psychomotor agitation associated with altered consciousness following anesthesia [1].

Inflammation and oxidative stress are thought to play a significant role in its pathophysiology. Several studies have demonstrated an association between neutrophil-to-lymphocyte ratio (NLR) and delirium, particularly in elderly internal medicine patients and cases of ischemic stroke [2,3]. In patients undergoing head and neck flap surgery who are monitored in the postoperative intensive care unit, as well as those undergoing vascular surgery and esophagectomy, delirium rates have been found to correlate with preoperatively assessed NLR [4,5,6].

The incidence of postoperative delirium in children aged 3 to 7 years undergoing adenoidectomy and tonsillectomy has been reported to be approximately 25.3% [7]. The rate is higher in young children and preschool-aged children. The underlying mechanism for the high incidence of delirium in this young age group remains unclear. However, various factors such as age and pre-existing anxiety, rapid emergence from sevoflurane anesthesia, hypoxemia, hypercapnia, sepsis, hypoglycemia, electrolyte imbalance, residual drug effects, and pain are known to contribute to the development of delirium [1]. Understanding risk factors is essential for prevention, accurate diagnosis, and effective treatment. In recent years, predicting the occurrence of postoperative delirium by specific biomarkers has become a prominent area of research. The aim of this study is to investigate the relationship between delirium and inflammatory markers—namely, neutrophil-to-lymphocyte ratio (NLR), mean platelet volume (MPV), and platelet distribution width (PDW)—in children aged 3 to 9 years undergoing adenoidectomy and tonsillectomy.

## 2. Methods

This study was conducted in accordance with the Declaration of Helsinki, and the protocol was approved by the Ethics Committee of Ankara Bilkent City Hospital (No. E1-23-3197) on February 8, 2023. This single-center, prospective, and observational study included 221 children aged 3 to 9 years in the ASA 1–3 risk group who underwent adenoidectomy, tonsillectomy, and/or ventilation tube placement. Consent was obtained from the parents, and patients were either premedicated with oral midazolam (0.5 mg.kg^−1^) in the preoperative period or taken to surgery without premedication, depending on the anesthesiologist’s preference. After induction with sevoflurane, intravenous access was established, and fentanyl (1 µg.kg^−1^) and rocuronium (0.6 mg.kg^−1^) were administered. Following intubation, maintenance was achieved with sevoflurane, 50% oxygen, 50% air, and a remifentanil infusion at 0.1–0.15 µg.kg^−1^.min^−1^. Additionally, all children received intravenous paracetamol (15 mg.kg^−1^), methylprednisolone (1 mg.kg^−1^), and ondansetron (0.1 mg.kg^−1^). At the end of the operation, the muscle relaxant effect was reversed with sugammadex at 2 mg.kg^−1^. Following extubation, patients were transferred to the post-anesthesia recovery unit and monitored for 30 min in the presence of their parents. In the postoperative period, patients’ pain was assessed using the Face, Legs, Activity, Cry, and Consolability (FLACC) Scale, and the presence of delirium was evaluated with the Pediatric Anesthesia Emergence Delirium (PAED) Scale.

Self-assessment scales cannot be applied to the youngest age group of patients due to their inability to communicate verbally [8]. One of the most used behavioral scales is the FLACC Scale (Table 1). The FLACC Scale is used to assess the intensity of postoperative pain in young children, infants, or those unable to communicate, including children aged 2 months to 7 years who are asleep. Each parameter is assessed on a scale from 0 to 2, with the total score interpreted as follows: 0 = relaxed and comfortable; 1–3 = mild discomfort; 4–6 = moderate pain; 7–10 = severe discomfort/pain. A score greater than 3 indicates a need for analgesics [8,9]. In our study, a patient with a score higher than 3 was first treated with ibuprofen. After pain relief, the patient’s delirium was assessed by PAED Score.

The Pediatric Anesthesia Emergence Delirium (PAED) Scale is the only validated tool for measuring delirium and agitation in the postoperative period (Table 2). The PAED score is used to assess delirium after the patient awakens and prior to the administration of medication, to differentiate pain from delirium. Accordingly, the PAED score is determined by evaluating each category—eye contact, purposeful movements, awareness of surroundings, restlessness, and inconsolability—on a scale from 0 to 4. A total score equal to or greater than 10 indicates the presence of delirium [10]. In our study, all patients were assessed using the PAED scoring system in the postoperative period, and those with a score of 10 or higher were considered to have delirium.

Blood analysis was performed preoperatively under same conditions to all patients and complete blood count values were recorded.

### Statistical Analysis

Statistical analyses and data visualization were performed using R Studio version 2024.09.0+375 (“Cranberry Hibiscus”), running on macOS with Quarto version 1.5.57. (RStudio Team (2020), Integrated Development for R. RStudio, PBC, Boston, MA, USA, http://www.rstudio.com/, accessed on 15 August 2023). In our descriptive analyses, quantitative data were expressed as mean and standard deviation, while qualitative categorical variables were presented as case numbers and percentages (%). In the analysis of NLR levels in patients classified as having delirium, tests for normal distribution and homogeneity of variance were conducted to ensure the assumptions for the Student’s *t*-test were met. Shapiro–Wilk normality tests were performed for the “Yes” and “No” groups. The Student’s *t*-test was used to compare the mean NLR levels between the two groups. The association between the need for analgesics and the presence of delirium in children was evaluated using the chi-squared test. 

## 3. Results

The demographic and clinical characteristics of the patients are presented in Table 3. Regarding gender distribution, 58% of the patients were male, and 42% were female. No comorbidities were present in 91.4% of the patients, while only 8.6% had comorbidities. Comorbidities of the 19 patients were 11 allergic bronchitis, 1 ventricular septal defect, 1 obesity, 1 gluten enteropathy, 1 eczema, 1 febrile convulsion, 1 cerebral palsy, 1 situs inversus, and 1 mitral insufficiency. The distribution of ASA scores indicated that many participants (91.9%) were in the low-risk category (ASA 1), with a minimal proportion at high risk (0.45% in ASA 3). In terms of types of surgery, adenoidectomy (53.4%) and adenotonsillectomy (42.5%) were the most common procedures, showing that these two types predominated within the study population. The average durations of surgery and anesthesia were reported as 64 and 75 min, respectively, with standard deviations reflecting the variation in these durations. Finally, with respect to nausea and vomiting, 92.8% of participants did not experience these side effects, whereas only 7.2% reported nausea and vomiting. Premedication was administered to 38% of the patients.

The cross-tabulation provides a detailed presentation of the relationships between delirium and associated hematologic parameters (Table 4). In our study, delirium was observed in 36.2% of patients. No significant differences were found between patients with and without delirium in terms of gender, duration of anesthesia, or presence of comorbidities. Similarly, when comparing groups based on whether premedication was administered, no statistically significant difference was identified. The table shows that hematologic parameters including MPV, PDW, NLR, lymphocyte-to-monocyte ratio (LMR), neutrophil-to-monocyte ratio (NMR), platelet-to-lymphocyte ratio (PLR), neutrophil, and platelet values were similar between patients with and without delirium. Lymphocyte count, PAED score, and FLACC score were found to be statistically significantly higher in individuals with delirium compared to those without delirium (*p* < 0.005). The Shapiro–Wilk test for normality indicated that the conditions for normal distribution were met for both groups (delirium present and delirium absent). The *p*-values were 0.0003167 for the delirium-present group and 9.79 × 10^−5^ for the delirium-absent group. A two-sample *t*-test showed a significant difference in the mean FLACC scores between the two groups (t = 21.372, df = 169.87, *p*-value < 2.2 × 10^−16^). In contrast, the FLACC scores themselves did not follow a normal distribution within either group (Shapiro–Wilk *p*-value = 0.1612 for the delirium-present group and 0.4298 for the delirium-absent group). A Wilcoxon rank-sum test confirmed a significant difference in PAED and FLACC scores between the two groups (W = 0, *p*-value < 2.2 × 10^−16^ and W = 9489, *p*-value < 2.2 × 10^−16^, respectively).

Another noteworthy finding is the need for analgesics. While 91% of patients with delirium required analgesics, this rate was 47% among those without delirium. The difference was found to be statistically significant (X-squared = 41.313, df = 1, *p*-value = 1.297 × 10^−10^). Finally, lymphocyte levels and not the ratios including lymphocytes were significantly different between the two delirium statuses (W = 4407.5, *p*-value = 0.006997).

### Regression Models 

We constituted Directed Acyclic Graphs to decide the covariables to be included in this study to stay cautious for multicollinearity and over-fitting. Yet, in our first logistic regression model examining factors affecting the risk of delirium in the postoperative period, various results were obtained regarding biomarkers and demographic variables for exploratory reasons; these models are not included in the final table. Variables such as NLR, LMR, NMR, and PLR did not generally show a significant effect on the risk of delirium. Following supplementary analyses, we discovered that PLR and NMR values were highly correlated with NLR values (coefficients of correlation 0.85 and 0.87, respectively), so we decided to retract these variables in the models. A lack of evidence regarding the tangible impact of other variables such as PDW and receiving premedication drove us to exclude these variables for our final model (Table 5).

The OR value of 0.97 for NLR suggests a potential 3% reduction in the risk of delirium per unit increase, but this result is not statistically significant (*p* = 0.659). This lack of significance, coupled with a confidence interval that crosses 1, indicates that the observed effect could very well be due to random variation rather than a meaningful association. On the other hand, age emerged as a strongly significant predictor, with each additional year associated with a 46% increase in the risk of delirium (OR = 1.46, *p* < 0.001). The stark contrast between the statistical importance of age and the ambiguity surrounding NLR underscores the limited predictive utility of NLR in this context, particularly when adjusting for other covariates. The Likelihood Ratio Test comparing Model 1 (including only NLR and age) and Model 2 (including NLR, age, weight, gender, and comorbidities) indicates that the addition of weight, gender, and comorbidities does not significantly improve the model’s fit (*p* = 0.656). The small deviance difference (1.6164) and the high *p*-value suggest that these additional variables do not contribute meaningfully to explaining the variability in the risk of delirium beyond what is already accounted for by NLR and age. This result supports the use of the simpler Model 1, as it provides a similarly effective fit to the data without the complexity introduced by the additional covariates.

Furthermore, there is a moderate to strong positive correlation between average PAED and FLACC scores (*r* = 0.652, 95% CI [0.569, 0.722], *p* < 0.001), indicating a significant relationship between the two measures. Since these two show two different dimensions of the same phenomena, although closely related, we decided to include FLACC score in another exploratory model. 

The inclusion of FLACC mean score as a variable in the logistic regression model significantly improved its explanatory power. The results indicate that FLACC mean score is a strong and independent predictor of delirium, with an adjusted OR of 2.52 (95% CI [1.94, 3.28], *p* < 0.001). This suggests that higher FLACC scores are associated with a substantially increased risk of delirium. In contrast, the effect of NLR remained non-significant after adjusting for FLACC (aOR = 1.02, 95% CI [0.78, 1.33], *p* = 0.878), reinforcing its limited role as a predictor in the presence of this variable. Age retained its significance, with an adjusted OR of 0.66 (95% CI [0.48, 0.90], *p* = 0.008), suggesting that older age is associated with a reduced risk of delirium in this context. The addition of FLACC mean score significantly improved the model fit, as indicated by the reduction in AIC. The AIC for the model without FLACC was 285.32, whereas the AIC for the model including FLACC decreased to 209.84.

## 4. Discussion

In this study investigating the potential relationship between inflammatory markers and postoperative delirium, no association was found between hematologic biomarkers such as NLR, LMR, NMR, PLR, MPV, and PDW and the occurrence of delirium. Additionally, variables such as weight, gender, and the administration of premedication did not show a significant effect on the risk of delirium.

Delirium is a fluctuating state of consciousness that occurs during the recovery period following anesthesia, accompanied by perceptual disturbances, hallucinations, complex behavioral disorders, and psychomotor agitation [11]. Sikich and Lerman defined postoperative delirium as ‘a disturbance in the child’s awareness of and attention to their surroundings, accompanied by disorientation and perceptual changes, including hypersensitivity to stimuli and hyperactive motor behavior immediately following anesthesia’ [10]. It typically occurs within the first 30 min after emergence from anesthesia, is self-limiting (5–15 min), and generally resolves spontaneously. However, agitation and regressive behaviors lasting up to 2 days have also been described. Factors such as rapid emergence, characteristics of the anesthetic agent, postoperative pain, type of surgery, age, preoperative anxiety, the child’s temperament, and concomitant medications have been implicated [11].

During this period of disorientation, the patients may harm themselves by removing intravenous catheters, drains, and dressings, leading to prolonged discharge time and reduced patient and family satisfaction [11]. For these reasons, early diagnosis of delirium is important.

The prevalence of postoperative delirium can vary between 10% and 80% in all children receiving general anesthesia [11]. Otolaryngologic surgeries have been shown to increase the risk [12]. The prevalence of postoperative delirium in children aged 3 to 7 years undergoing adenoidectomy and tonsillectomy was found to be 25.3% [7]. In the retrospective study conducted by Somaini et al. on 512 children, the incidence of delirium was found to be 69%, with both delirium and pain occurring together in 15% of the children [13]. In the retrospective study by Liu et al., which evaluated records of 4974 patients aged 18 and under who underwent tonsillectomy and adenoidectomy, the incidence of delirium was found to be 1.3%. This rate is lower than the results reported in other studies in the literature. The prevalence was higher in the 1–3 age group (2.9%) and among male children (1.6%). ASA score and duration of anesthesia showed no difference between the groups with and without delirium [14]. In our study, delirium was observed in 36.2% of patients, consistent with the rates reported in the literature. In our study, FLACC and PAED scores were found to be significantly higher in patients with delirium, suggesting that delirium may be an important indicator in assessing pain and comfort levels. Among the total of 221 patients, 73 (33.33%) experienced both delirium and pain. In terms of analgesic need, 91% of patients with delirium required analgesics, whereas this rate was 47% in those without delirium. This may indicate that patients with delirium experience more pain or perceive pain more intensely. No clear conclusion could be reached regarding whether clinical parameters, such as the need for analgesics and type of surgery, are directly related to delirium.

Early diagnosis of delirium is crucial, and the search for diagnostic and prognostic biomarkers to aid clinicians in this regard is ongoing. Research in this area is highly limited, and the results are often contradictory. Inflammatory markers have been associated with delirium in studies; however, their use in clinical practice has not been practical due to cost and the complexity of the diagnostic process. Simple laboratory tests, which are readily available in every center and performed at the time of admission for all patients, are currently under consideration [3].

The underlying pathophysiology of delirium is not fully understood; however, inflammation and oxidative stress are suggested to play key roles. The NLR is an easily accessible marker of inflammation and oxidative stress that reflects the balance between neutrophils and lymphocytes. It can be readily obtained from the initial complete blood count at hospital admission and is less affected by fluid imbalances compared to white blood cell subgroups [2]. An elevated NLR value has a stronger predictive value compared to traditional inflammatory markers such as CRP and white blood cell count [2]. Additionally, it is more predictive than CRP, neutrophil, and lymphocyte values individually [15]. However, there is a limited number of studies examining the relationship between NLR and delirium.

NLR is used in prognostic monitoring for various conditions such as malignancy, cardiovascular disease, and sepsis [16,17,18]. Additionally, some studies have reported an association between elevated NLR levels and neuropsychiatric conditions such as Alzheimer’s disease, schizophrenia, Parkinson’s disease, ischemic stroke, and memory disorders [3,19,20].

In terms of postoperative delirium, higher preoperative NLR has been shown to be associated with the development of postoperative delirium in patients undergoing cardiac surgery [21], carotid endarterectomy [22], total hip arthroplasty [23], vascular surgery [5], head and neck free-flap surgery [4], gastrointestinal and genitourinary surgery [24], and esophagectomy [6]. In a recently published study by Feng et al., preoperative NLR and postoperative pain were reported as independent risk factors for postoperative delirium in pediatric patients aged 3 to 7 years undergoing tonsillectomy and adenoidectomy. The cut-off value for NLR was determined to be 0.8719 [7].

In contrast, Ida et al. found no significant association between preoperative NLR and postoperative delirium [25]. Geriatric patients undergoing elective abdominal surgery were included in the study and the relationship between hematological biomarkers and delirium was analyzed. There was no statistically significant difference in preoperative NLR in the patients who experienced postoperative delirium. Patients with delirium had larger blood loss volumes and longer durations of surgery. In another study carried out by Kato et al., the preoperative NLR of elderly patients undergoing degenerative spine surgery was not significantly associated with postoperative delirium, but a lower preoperative uric acid value was found to be an independent risk factor for developing delirium [26]. In a study conducted by Derin et al., preoperative and postoperative NLR levels were examined in 76 pediatric patients undergoing adenoidectomy. A preoperative and third-month postoperative complete blood cell count was performed to calculate the NLR values in all patients. The main finding of the study was the absence of a correlation between the preoperative and postoperative NLR. They observed that adenoidectomy does not cause a significant change in NLR levels and demonstrated that the NLR is not a statistically significant inflammatory factor. They reported that NLR could not be used for diagnostic or prognostic monitoring of upper airway obstruction [27]. In our study, like these previous studies, no association was found between postoperative delirium and hematologic inflammatory markers such as NLR, LMR, NMR, PLR, MPV, and PDW. The different results obtained among the studies can be attributed to patient characteristics, age, surgical procedures, duration of surgery, blood loss, different anesthetic agents, and different diagnostic tools for delirium.

In conclusion, demographic and clinical factors, along with potential biological markers, need to be considered to better understand and manage pediatric delirium. Multi-center, controlled studies with larger sample sizes are needed to determine whether hematologic markers such as NLR, LMR, NMR, PLR, MPV, and PDW have strong predictive value.

Our study has several limitations: Firstly, only preoperative markers of the patients were examined, and postoperative complete blood counts were not evaluated. Secondly, there was no control group. The values of patients during their healthy periods were not examined; therefore, their baseline values are unknown. Lastly, patients’ anxiety levels in the preoperative period were not assessed.

This study, which investigated the potential relationship between inflammatory markers and postoperative delirium, found that hematologic inflammatory parameters such as NLR, LMR, NMR, PLR, MPV, and PDW did not have a significant effect on delirium risk.

## Figures and Tables

**Table 1:** The FLAAC Scale [9].

Categories	Scoring		
	0	1	2
Face	No particular expression or smile	Occasional grimace or frown, withdrawn, disinterested	Frequent to constant frown, clenched jaw, quivering chin
Legs	Normal position or relaxed	Uneasy, restless, tense	Kicking, or legs drawn up
Activity	Lying quietly, normal position, moves easily	squirming, shifting back and forth, tense	Arched, rigid or jerking
Cry	No crying (awake or asleep)	Moans or whimpers, occasional complain	Crying steadily, screams or sobs, frequent complaints
Consolability	Content, relaxed	Reassured by occasional touching, hugging or being talked to, distractible	Difficult to console or comfort

Each of the 5 categories, (F) Face; (L) Legs; (A) Activity; (C) Cry; (C) Consolability, is scored 0–2, which results in a total score between 0 and 10.

**Table 2:** The PAED Scale [10].

1. The child makes eye contact with the caregiver. 2. The child’s actions are purposeful. 3. The child is aware of his/her surroundings. 4. The child is restless. 5. The child is inconsolable.

Items 1, 2, and 3 are reversed scored as follows: 4 = not at all; 3 = just a little; 2 = quite a bit; 1 = very much; 0 = extremely. Items 4 and 5 are scored as follows: 0 = not at all; 1 = just a little; 2 = quite a bit; 3 = very much; 4 = extremely. The scores of each item were summed to obtain a total Pediatric Anesthesia Emergence Delirium (PAED) scale score. The degree of emergence delirium increased directly with the total score.

**Table 3:** Demographic and clinical characteristics of study patients (N = 221).

Sociodemographic Characteristics	Total Number (n), Percentage (%)
**Age (years)**	5.3 ± 1.37
**Gender, n (%)**	
*Male*	128.0 (58.0)
*Female*	93.0 (42.0)
**Comorbidity, n (%)**	
*No*	202 (91.4)
*Yes*	19 (8.6)
**ASA Score, n (%)**	
*1*	203 (91.9)
*2 and higher*	18 (8.1)
**Surgery, n(%)**	
*Adenoid*	118 (53.4)
*Tonsil*	9 (4.1)
*Adenotonsil*	94 (42.5)
**Ventilation Tube Placement, n (%)**	
*No*	132 (60)
*Yes*	89 (40)
**Duration of Surgery (minutes)**	64 ± 21
**Duration of Anesthesia (minutes)**	75 ± 22
**Nausea—Vomiting, n (%)**	
*No*	205 (92.8)
*Yes*	16 (7.2)
**Premedication, n (%)**	
*No*	137 (62)
*Yes*	84 (38)

Continuous numerical variables are presented as mean and standard deviation, while categorical variables are presented as counts and percentages.

**Table 4:** Sociodemographic characteristics and comparison of studied variables (N = 221).

	Delirium Is Present N = 80 (36.2%)	Delirium Is Absent N = 141 (63.8%)	*p*-Value
**Gender, n (%)**			0.9
*Male*	47 (59)	81 (57)	
*Female*	33 (41)	60 (43)	
**Premedication, n (%)**			0.9
*Yes*	31 (39)	53 (38)	
*No*	49 (61)	88 (62)	
**Duration of Anesthesia**	72.30 ± 18.65	76.94 ± 23.68	0.2
**Comorbidity, n (%)**			0.3
*No*	75 (94)	127 (90)	
*Yes*	5 (6.3)	14 (9.9)	
**PDW**	41.78 ± 6.82	41.15 ± 7.63	0.131
**MPV**	7.59 ± 0.64	7.69 ± 0.73	0.496
**Neutrophil**	4.56 ± 2.32	4.34 ± 1.91	0.057
**Lymphocyte**	3.67 ± 1.18	3.29 ± 1.12	***<0.005***
**Monocyte**	0.45 ± 0.16	0.43 ± 0.13	0.341
**Platelet**	369.39 ± 81.62	356.31 ± 87.39	0.265
**NLR Ratio**	1.61 ± 2.35	1.61 ± 1.75	0.189
**LMR Ratio**	8.67 ± 3.41	8.15 ± 3.04	0.242
**NMR Ratio**	10.54 ± 6.29	10.70 ± 5.96	0.457
**PLR Ratio**	113.66 ± 63.31	121.50 ± 61.73	0.140
**Mean PAED Score**	12.20 ± 1.71	7.02 ± 1.78	***<0.005***
**Mean FLACC Score**	5.18 ± 1.56	3.02 ± 1.45	***<0.005***
Analgesics need *, n (%)			***<0.005***
*Yes*	73 (91,25)	66 (47)	
*No*	7 (8.75)	75 (53)	

Continuous numerical variables are presented as mean and standard deviation, while categorical variables are presented as counts and percentages. * The analysis was conducted using a chi-squared test. PDW: platelet distribution width; MPV: mean platelet volume; NLR: neutrophil-to-lymphocyte ratio; LMR: lymphocyte-to-monocyte ratio; NMR: neutrophil-to-monocyte ratio; PLR: platelet-to-lymphocyte ratio.

**Table 5:** Association between NLR ratio and delirium odds: results from multivariable logistic regression analyses (Turkey, 2024).

Variables	Model 1: aOR (95% CI)	*p*-Value	Model 2: aOR (95% CI)	*p*-Value
**NLR (continuous)**	1 (0.87, 1.15)	0.659	1.03 (0.89, 1.19)	0.675
**Age (continuous)**	0.69 (0.55, 0.86)	<0.001	0.68 (0.52, 0.88)	<0.05

Model 1 (bivariate): Adjusted logistic regression model including NLR and age as predictors for postoperative delirium. Model 2 (multivariate): Adjusted logistic regression model including NLR, age, weight, gender, and comorbidities as covariates to evaluate their independent contributions to the risk of delirium.

## Data Availability

The data presented in this study are available on request from the corresponding author.
